# Ups and downs in the experimental treatment of murine typhoid fever using gentamicin combined with caffeine

**DOI:** 10.1007/s42770-026-01948-2

**Published:** 2026-04-27

**Authors:** Esther de Souza Silva, Roseane Thays dos Santos Rocha, Joyce Nayara Gomes da Silva, Victor Vinicius Pereira Ribeiro, Islana Silva Ponte, Isabela Cristina Bandeira Fraga, Camila Sant Ana de Oliveira, Viviane Moreira Lima Galvão, Alison Hideo Jolo Silva, Valdemiro Amaro da Silva Junior, José Vitor Lima-Filho

**Affiliations:** 1 Department of Biology, Federal Rural University of Pernambuco, Recife, PE Brazil; 2 Department of Veterinary Medicine, Federal Rural University of Pernambuco, Recife, PE Brazil; 3 Laboratório de Microbiologia e Imunologia, Departamento de Biologia, Universidade Federal Rural de Pernambuco, Dom Manoel de Medeiros, s/n, B. Dois Irmãos, Recife, PE CEP 52171-900 Brasil

**Keywords:** *Salmonellatyphimurium*, Immunomodulation, Therapeutic adjuvant

## Abstract

This study assessed whether combining the aminoglycoside gentamicin with caffeine enhances antimicrobial efficacy in a murine typhoid model. We infected murine peritoneal macrophages in vitro with *Salmonella enterica* serovar Typhimurium and treated them for 24 h with non-toxic concentrations of caffeine (0.05–5 µg/ml) plus gentamicin (10 µg/ml). Subsequently, Swiss mice were orally challenged with *S.* Typhimurium and received daily intraperitoneal doses of caffeine (0.05-5 mg/kg) plus gentamicin (10 mg/kg) for five days post-infection. Macrophages treated with 5 µg/ml caffeine plus 10 µg/ml gentamicin exhibited significantly higher viability and a marked reduction in intracellular bacterial load. Lower caffeine doses (0.05 and 0.5 µg/ml) failed to preserve cell viability due to uncontrolled bacterial proliferation. Mice receiving gentamicin – either alone or combined with 5 mg/kg caffeine – showed reduced hepatic bacterial burdens and milder histopathological damage. Notably, only the higher caffeine dose enhanced gentamicin’s efficacy; lower caffeine concentrations antagonized the antibiotic’s activity.

## Introduction

According to the Brazilian Ministry of Health, 6,874 outbreaks of water and foodborne diseases (WFDs) were recorded from 2014 to 2023 [1] However, the etiological agents were identified in only 1,644 cases, 9.6% of which were caused by *Salmonella* species. The *Salmonella* genus comprises Gram-negative, facultatively anaerobic bacteria with rod-like morphology and peritrichous flagella of two species: *Salmonella enterica* and *S. bongori* [2]. Both species are pathogenic, but *S. enterica* is more relevant to public health, having approximately 2,600 serotypes, several of which can affect humans and animals [3, 4]. For instance, human typhoid fever, caused by *S. enterica* serovar Typhi, is a global disease with high rates of hospitalization and mortality [5, 6]. In murine models, *Salmonella enterica* subspecies enterica serovar Typhimurium (*S*. Typhimurium) causes symptoms analogous to human typhoid fever and is widely used as an animal model of the disease. After ingestion of contaminated food or water, the bacterium invades M cells in the intestinal epithelium and reaches the lamina propria [7]. In Peyer’s patches, the pathogen is phagocytosed by macrophages and can multiply within membrane-bound inclusions named *Salmonella*-containing vacuoles (SCVs) [8]. The release of proinflammatory cytokines recruit neutrophils to the site of infection and recirculates antigen-presenting cells to the mesenteric lymph nodes [9]. Intra- and extracellular bacterial replication leads to systemic dissemination in the bloodstream and the emergence of new infectious foci, such as the spleen and liver [10, 11].

Treatment for disseminated *Salmonella* infections typically includes fluoroquinolones, cephalosporins, macrolides, and carbapenems. Fluoroquinolones (e.g., ciprofloxacin) inhibit DNA replication and are the first-line therapy, although resistant strains have been reported [12]. Cephalosporins (e.g., ceftriaxone) impair cell-wall synthesis and are favored against resistant isolates [13]. Macrolides (e.g., azithromycin) block protein synthesis and serve as alternatives for resistant cases [14]. Carbapenems, which also target cell‐wall synthesis, are reserved for severe, multidrug‐resistant infections [15]. Gentamicin is a broad-spectrum aminoglycoside that inhibits protein synthesis [16]. The antibiotic is not routinely used to treat salmonellosis, but it is commonly employed to manage invasive Gram-negative infections [17]. The World Health Organization recommends gentamicin, along with ampicillin, for the treatment of neonatal sepsis [18]. Although gentamicin is very effective against extracellular bacteria, its role against intracellular bacteria is limited. Menashe [19] has shown that concentrations between 15 and 150 µg/ml do not kill intracellular *S.* Typhimurium. However, the combination of gentamicin with different anti-infective drugs can potentially increase the range of therapeutic choices against salmonellosis.

Previously, we demonstrated that caffeine, despite not having direct antimicrobial activity, enhanced the bactericidal capacity of macrophages infected with *S.* Typhimurium [20]. Caffeine (C_8_H_10_N_4_O_2_) is classified as an alkaloid belonging to the methylxanthine class (1,3,7-trimethylxanthine) that is present in natural products such as coffee beans (*Coffea arabica*), tea leaves (*Camellia sinensis*), guarana seeds (*Paullinia cupana*), cocoa seeds (*Theobroma cacao*), mate leaves (*Ilex paraguariensis*), cola seeds (*Cola nitida*) among others [21, 22]. It is well recognized as a pharmacologically active molecule and has been extensively studied for its central nervous system–stimulant potential. Caffeine is often included in pharmaceutical products as an analgesic adjuvant in over-the-counter formulations for mild pain, typically combined with dipyrone, ibuprofen, acetylsalicylic acid, and other drugs. As an adjuvant, caffeine has no direct analgesic action but enhances the efficacy of the drug with which it is combined [23] although its potential in antimicrobial therapies remains underexplored. The aim of the present study was to evaluate the therapeutic potential of combining gentamicin with caffeine in a murine typhoid fever model.

## Materials and methods

### Animals

Swiss mice of both sexes, aged 4 to 6 weeks and weighing 35 to 40 g, were obtained from the Animal Facility of the Keizo Asami Immunopathology Laboratory (LIKA) at the Federal University of Pernambuco, Brazil. During the experiments, the animals were kept in the Animal Facility of the Microbiology and Immunology Laboratory (LAMIM - UFRPE), under a 12-hour light/dark cycle, at a temperature between 22 and 23 °C. Water and food were offered *ad libitum*. Assays with murine peritoneal macrophages and Swiss mice were adapted from Tavares et al. [24]. All animal experiments were approved by the Committee on Ethical Use of Animals of Federal Rural University of Pernambuco (CEUA No. 7845120923) following international guidelines.

### Caffeine and gentamicin

**Fig. 1:**
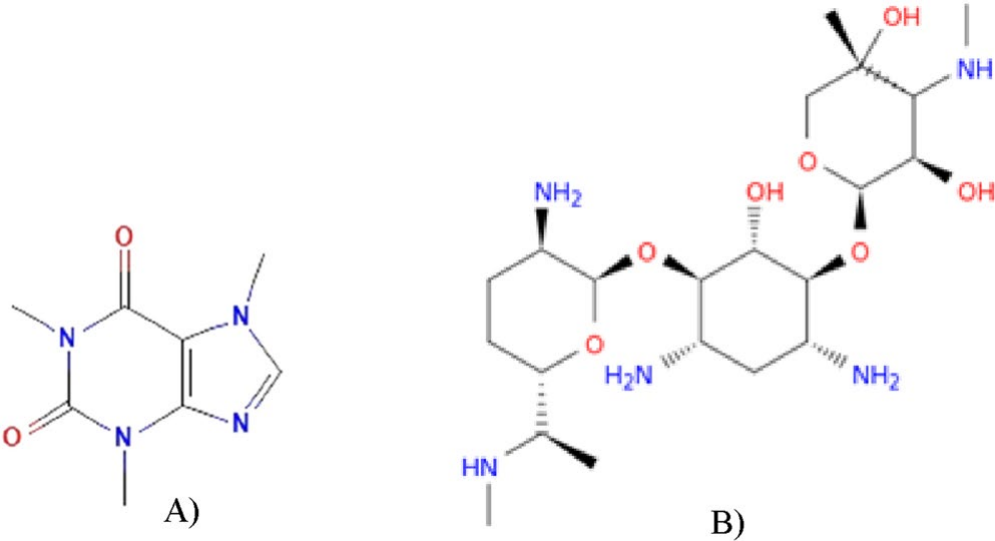
Chemical structures of caffeine (**A**) and gentamicin (**B**)

Caffeine (CAF) (C₈H₁₀N₄O₂; 194.19 g/mol – Lot BCBV8010) was commercially purchased as a powder (Sigma-Aldrich, Merck, St. Louis, USA) and dissolved in phosphate-buffered saline (PBS) pH 7.4 (NaCl 130 mM + Na_2_HPO4 7 mM + NaH_2_PO_4_ 3 mM) to produce stock solutions of 2 mg/mL. Gentamicin (GEN) (C₂₁H₄₃N₅O₇; 477.5 g/mol – Lot 398578) was commercially purchased (Sigma-Aldrich, Merck, St. Louis, USA) as a solution of 50 mg/mL (Fig. 1).

### Microorganism

*Salmonella enterica* ser. Typhimurium (strain C5) or simply *S*. Typhimurium C5 is a wild type strain worldwide used as a model of murine typhoid fever. The bacterium is highly virulent with a lethal dose for 50% (LD_50_) by the intravenous route of infection of < 10 colony-forming units (CFU) in BALB/c mice [25]. The strain is sensitive to majority of antibiotics and particularly to gentamicin [26]. The bacterium was cryopreserved at −20 °C in a Mueller Hinton broth solution with 30% glycerin. To reactivate the strain, an aliquot of the bacterial suspension was added to Brain Heart Infusion (BHI) culture medium and incubated overnight at 37 °C. For use in the assays, the culture was adjusted to the desired inoculum in a spectrophotometer at 630 nm (Optical density (OD) 0.5 approx. 1 × 10^8^ Colony forming units (CFU)/ml).

### In vitro antibacterial activity assay

The broth microdilution method was adapted from the Clinical and Laboratory Standards Institute (CLSI), CLSI Document M07-A9 [27]. Solutions containing 0.2 ml of caffeine at concentrations ranging from 0.05 to 5 µg/ml combined with 10 µg/ml gentamicin in Mueller-Hinton broth (MH) (OXOID, Basingstoke, UK) were added to 96-well polystyrene plates. *S.* Typhimurium was then added to the wells (5 × 10^4^ CFU/well). Wells without treatment or containing only gentamicin (10 µg/ml) were used as controls. After 24 h of incubation at 37 °C, absorbance was read on a spectrophotometer with an optical density of 630 nm.

### Cell culture procedures

After euthanasia by isoflurane inhalation, the peritoneal cavity of female Swiss mice was injected with 5–10 ml of Roswell Park Memorial Institute (RPMI) medium (Sigma-Aldrich/Merck, USA). Peritoneal fluid was then collected, and cell viability was determined by the trypan blue exclusion method. Cell suspensions were adjusted to contain 1 × 10^6^ cells/ml in RPMI medium and added to 96-well plates. After overnight incubation with 5% CO_2_ at 37 °C, nonadherent cells in the supernatant were discarded. Primary peritoneal macrophages in the wells were characterized by optical microscopy and then used in the experiments.

### Cytotoxicity assay with murine peritoneal macrophages (pMØ)

To evaluate the influence of the proposed treatments on cell viability, murine pMØ were exposed to different combinations of caffeine (0.05; 0.5 and 5 µg/ml) plus gentamicin (10 µg/ml). Wells with pMØ cultured with RPMI alone (negative toxicity control) or exposed to 50% dimethyl sulfoxide (DMSO) (positive toxicity control) were used as controls. After 24 h of incubation with 5% CO_2_ at 37 °C, 20 µL of resazurin (0.15 mg/ml) dissolved in a sterile Phosphate-Buffered Saline (PBS) solution was added to the wells. Then, the plate was again incubated under the same conditions described above for a period of 1–4 h to evaluate cell metabolic activity through reduction of resazurin (blue) to resorufin (pink) in a microplate spectrophotometer at 570 and 600 nm. The results were expressed as a percentage relative to the cell viability of the pMØ not submitted to treatments. The assays were performed at least three times with technical triplicate.

### Effect of gentamicin combined with caffeine on murine peritoneal macrophages infected with *S.* Typhimurium

The pMØ were exposed to *S.* Typhimurium (2 × 10^6^ CFU/well) for 4 h in 96-well plates incubated under 5% CO_2_ at 37 °C. The supernatant was then removed, and the cells were washed with RPMI. Next, 0.2 ml of 100 µg/ml gentamicin was added to eliminate extracellular bacteria. After 1 h of incubation under 5% CO_2_ at 37 °C, the supernatant was removed and murine pMØ were exposed to different combinations of caffeine (0.05; 0.5–5 µg/ml) plus gentamicin (10 µg/ml) for 24 h. Wells with uninfected pMØ cultured with RPMI alone or infected with *Salmonella* without treatment were used as controls. The viability of cultured pMØ of all groups was assessed as described in 2.6. The results were expressed as a percentage relative to the cell viability of untreated/uninfected pMØ. To assess the influence of treatments on the bactericidal capacity of macrophages, the supernatant was removed, and 0.2 ml of Triton X-100 (0.5%) was added to the wells. The plates were incubated at 10 °C for 20 min to lyse the pMØ and release the intracellular bacteria. The cell lysate was then serially diluted and plated on BHI agar and Salmonella-Shigella (SS) agar plates using the drop plate method. The number of colony forming units (CFU) was accessed after overnight incubation at 37 °C. The assays were performed at least three times with technical triplicate.

### Effect of gentamicin co-administered with caffeine in Swiss mice infected *S.* Typhimurium

To produce a disseminated infection, male Swiss mice (*n* = 4/group) were fasted for 8 h before oral administration of *S.* Typhimurium (0.2 ml; 2 × 10^5^ CFU/mouse). Treatments with different combinations of caffeine (0.05; 0.5 or 5 mg/kg) plus gentamicin (10 mg/kg) started 30 min after the infectious inoculum and were administered daily, intraperitoneally, until the fourth day post-infection. Mice administered with phosphate saline (PBS) or only gentamicin were used as controls. Total and differential leukocyte quantification was performed on blood samples before euthanasia by isoflurane inhalation, on the fifth day after infection for all animal groups. For total leukocyte counts, 20 µL of blood was homogenized with 380 µL of Turk’s reagent, and an aliquot was then quantified using a Neubauer chamber under a light microscope. Slides with blood smears were stained with the Rapid Panoptic kit for differential leukocyte counts. For bacterial quantification, spleen and liver fragments were macerated and homogenized with PBS. The homogenized samples, in addition to the blood samples, were subjected to serial decimal dilutions. The number of CFUs was accessed as described in 2.7. Liver fragments were collected for histopathological examination. The tissue sections were mounted on glass slides and stained with hematoxylin and eosin (HE) and were subsequently analyzed by a specialist who was unaware of the treatments received by the animal groups.

### Statistics

The statistical differences between the groups were obtained by analysis of variance (ANOVA) followed by the Bonferroni test, with a confidence interval of *p* < 0.05. Analyses and corresponding graphs were constructed with GraphPad Prism version 10.3.1.

## Results

**Fig. 2:**
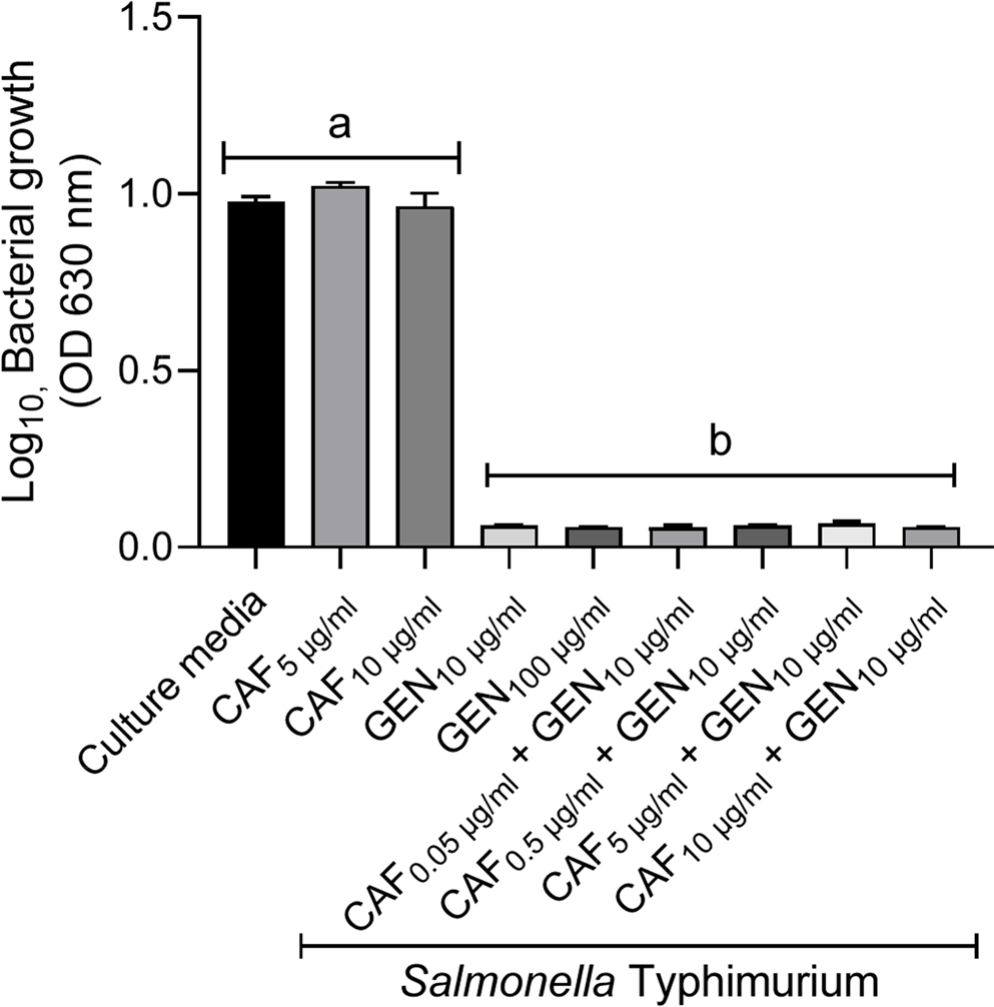
*In vitro* antimicrobial activity assay. Different superscript letters mean statistical differences with *p* < 0.05 (one-way ANOVA followed by Bonferroni’s post test). CAF – caffeine; GEN – gentamicin

**Fig. 3:**
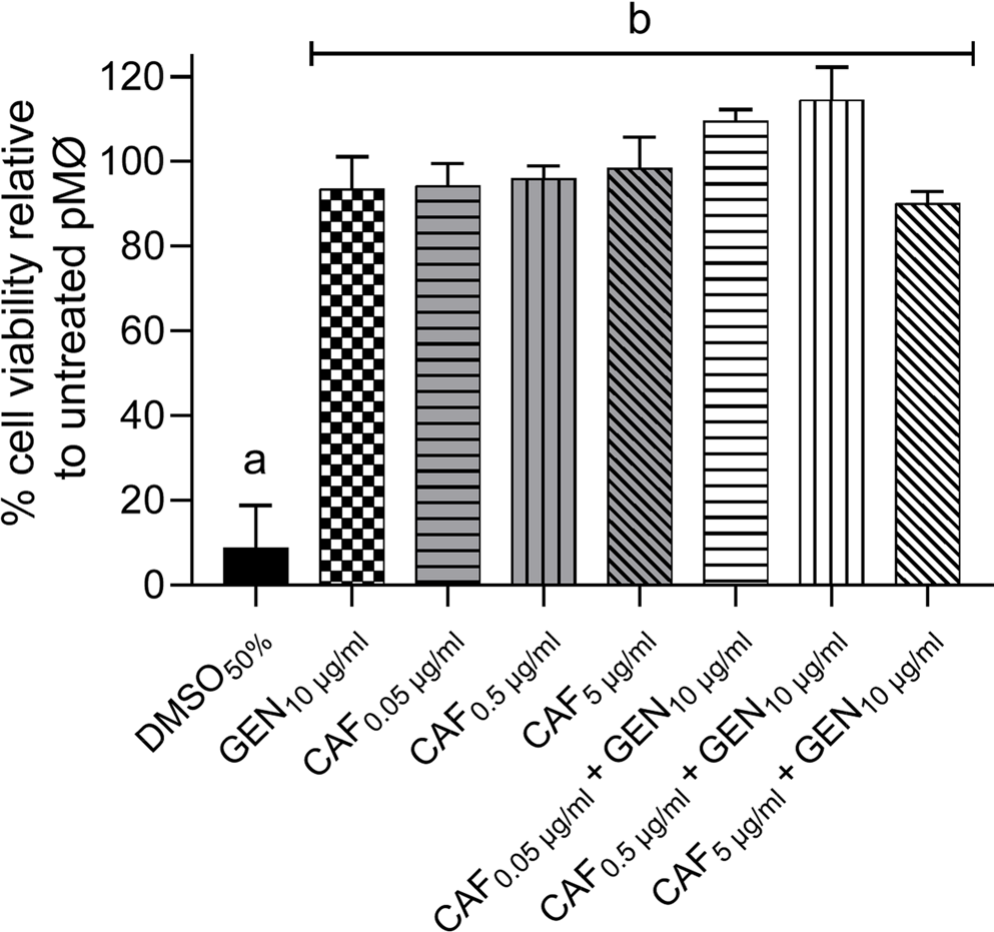
Viability of peritoneal macrophages treated with caffeine and/or gentamicin for 24 hours. Different superscript letters mean statistical differences with p < 0.05 (one-way ANOVA followed by Bonferroni’s post test). CAF – caffeine; GEN – gentamicin

**Fig. 4:**
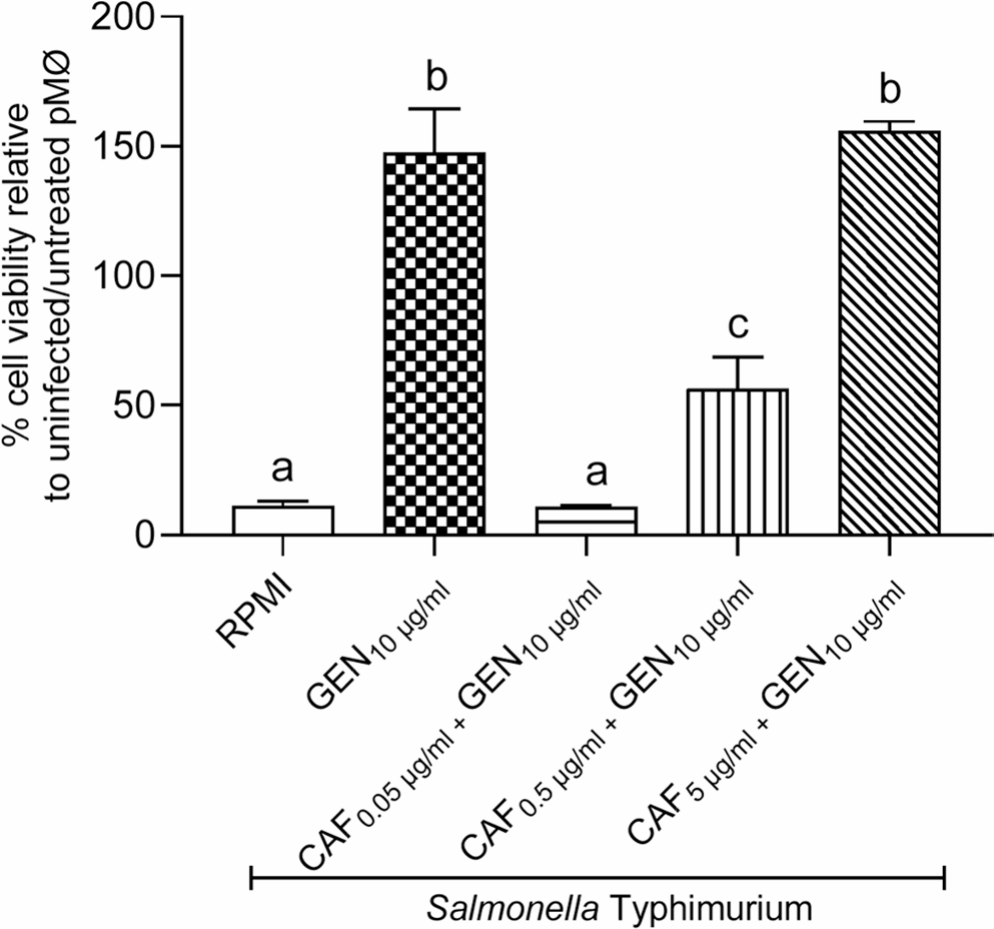
Viability of peritoneal macrophages infected with *Salmonella* Typhimurium and treated with caffeine and gentamicin. Different superscript letters mean statistical differences with p < 0.05 (one-way ANOVA followed by Bonferroni’s post test). CAF – caffeine; GEN – gentamicin

**Fig. 5:**
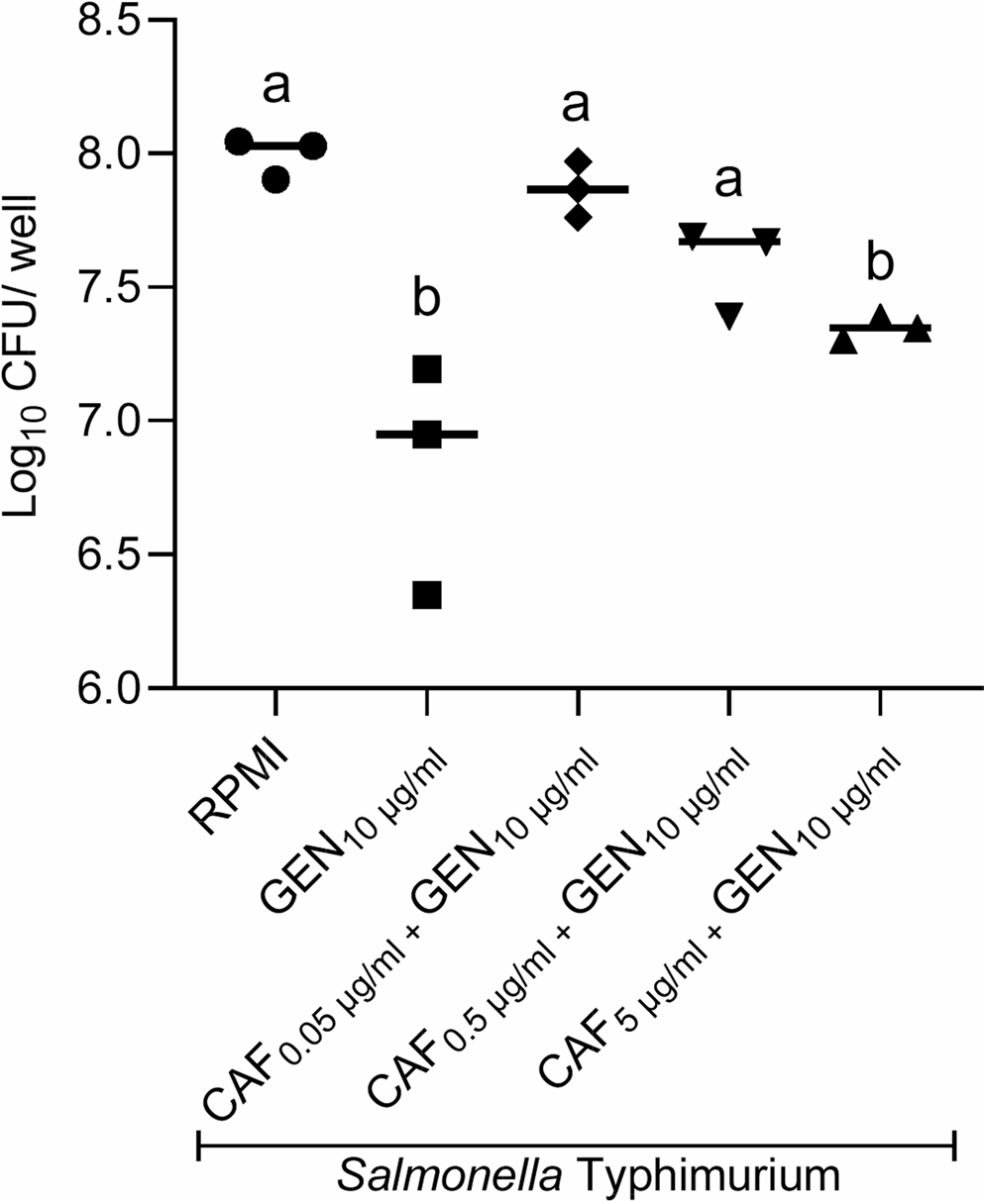
Intracellular bacterial quantification in murine peritoneal macrophages infected with *S.* Typhimurium and treated with caffeine and gentamicin for 24 hours. Different superscript letters mean statistical differences with p < 0.05 (one-way ANOVA followed by Bonferroni’s post test). CAF – caffeine; GEN – gentamicin

We confirmed that caffeine showed no direct antibacterial activity against *S*. Typhimurium C5, whereas gentamicin alone or combined with caffeine inhibited bacterial growth as expected (Fig. 2). These tested concentrations did not significantly alter the viability of cultured mouse peritoneal macrophages and were therefore not cytotoxic (Fig. 3). Assays with infected macrophages showed *S.* Typhimurium reduced cell viability in untreated pMØ (RPMI group), but also in macrophages treated with 0.05–0.5 µg/ml caffeine combined with 10 µg/ml gentamicin (Fig. 4). On the other hand, pMØ treated with gentamicin alone (10 µg/ml) or combined with caffeine (5 µg/ml), exhibited high cell viability, comparable to that of uninfected and untreated macrophages (Fig. 4). Within 24 h post-infection, these combined concentrations of gentamicin (10 µg/ml) and caffeine (5 µg/ml) led to a significant reduction in intracellular bacterial load compared to the untreated control group (*p* < 0.05) (Fig. 5). However, when reduced concentrations of caffeine were used (0.05 and 0.5 µg/ml), the bacterial load was high, despite gentamicin being maintained at the same dosage (10 µg/ml) (Fig. 5).

**Fig. 6:**
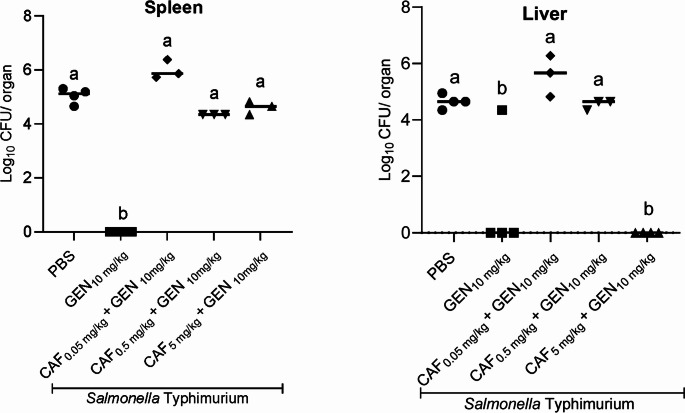
Bacterial quantification in target organs of *S*. Typhimurium infection following treatment with caffeine combined with gentamicin. Different superscript letters mean statistical differences with p < 0.05 (one-way ANOVA followed by Bonferroni’s post test). CAF – caffeine; GEN – gentamicin

Swiss mice were orally infected with *S.* Typhimurium C5 to induce a disseminated infection and simulate murine typhoid fever. However, following infection, some animals did not survive to the end of the experiment (Table 1). Daily treatment with gentamicin (10 mg/kg) co-administered with caffeine (5 mg/kg) successfully reduced bacterial presence in the liver five days post-infection, but not in the spleen (Fig. 6). Lower doses of caffeine (0.05 or 0.5 mg/kg), when combined with gentamicin (10 mg/kg), failed to reduce the bacterial load in either target organ, which remained elevated and comparable to levels observed in untreated mice (PBS group) (Fig. 6).

**Table 1:** Deaths post-infection

Groups	No. deaths
PBS	0
CAF 5 mg/kg	0
GEN 10 mg/kg	1
GEN 10 mg/kg + CAF 0.05 mg/kg	2
GEN 10 mg/kg + CAF 0.5 mg/kg	0
GEN 10 mg/kg + CAF 5 mg/kg	1

Table 2 shows the total and differential leukocyte quantification in the blood of animals subjected to the treatments. Five days after infection, there was a significant reduction in the number of total leukocytes in the blood of mice treated with gentamicin alone (10 mg/kg) or co-administered with caffeine (0.5 and 5 mg/kg) plus gentamicin (10 mg/kg), when compared to the untreated group (PBS) (*p* < 0.05). Differential leukocyte counts showed that neutrophil numbers were especially reduced in the groups of animals subjected to treatments compared to untreated ones (*p* < 0.05). The quantification of lymphocytes, monocytes, eosinophils and basophils was similar between the experimental groups and the control group.

**Table 2:** Quantification of total and differential leukocytes in the blood of Swiss mice after oral infection with *Salmonella* Typhimurium

	Groups	Total leukocytes (x 10^3^/mm^3^)	Differential leukocyte count (x 10^3^/mm^3^)				
			Neutrophil	Lymphocyte	Monocyte	Eosinophil	Basophil
*Salmonella* Typhimurium	PBS	0.37 ± 0.06	0.26 ± 0.04	0.09 ± 0.03	0.02 ± 0.01	0.00 ± 0.00	0.00 ± 0.00
	GEN 10 mg/Kg	0.19 ± 0.04 *	0.14 ± 0.02 *	0.05 ± 0.02 #	0.00 ± 0.00	0.19 ± 0.05	0.00 ± 0.00
	CAF 5 mg/Kg	0.20 ± 0.09	0.06 ± 0.00 *	0.19 ± 0.09	0.01 ± 0.01	0.00 ± 0.00	0.00 ± 0.00
	CAF 0.05 mg/Kg + GEN 10 mg/Kg	0.11 ± 0.07	0.16 ± 0.05 *	0.03 ± 0.02 #	0.01 ± 0.01	0.00 ± 0.00	0.00 ± 0.00
	CAF 0.5 mg/Kg + GEN 10 mg/Kg	0.18 ± 0.02 *	0.12 ± 0.01 *	0.05 ± 0.01 #	0.00 ± 0.00	0.00 ± 0.00	0.00 ± 0.00
	CAF 5 mg/Kg + GEN 10 mg/Kg	0.13 ± 0.08 *	0.11 ± 0.00 *	0.05 ± 0.01 #	0.01 ± 0.00	0.00 ± 0.00	0.00 ± 0.00

ANOVA ONE WAY followed by the Bonferroni test. * *p* < 0.05, statistical difference in relation to the PBS group; # *p* < 0.05, statistical difference in relation to the group CAF 5 mg/Kg

**Fig. 7:**
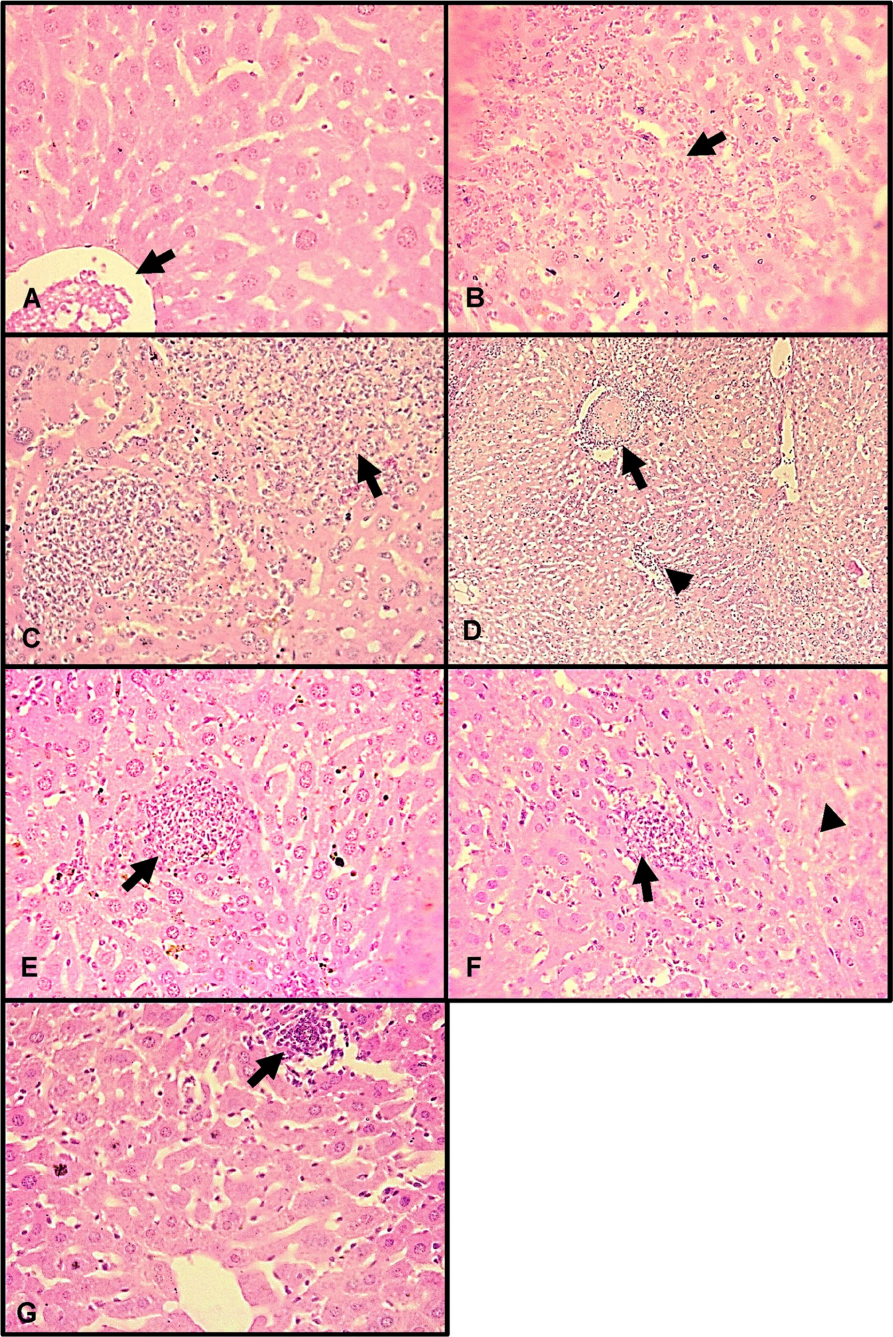
Histological liver damage in Swiss mice infected with *S.*Typhimurium and treated with gentamicin combined with caffeine. **A**) Uninfected/untreated group (400X; H&E). Note congested centrilobular veins (arrow); **B**) Infected/untreated group (400X; H&E). Note neutrophilic inflammatory focus (arrow); **C**) Infected group/GEN 10 mg/kg (400X; H&E). Note neutrophilic inflammatory foci (arrow) and inflammatory cells in the hepatic sinusoids; **D**) Infected group/CAF 5 mg/kg (100X; H&E). Note neutrophilic necrotic inflammatory focus (arrow) and mild to moderate inflammatory infiltrate among the sinusoidal capillaries (arrowhead); **E**) Infected group/CAF 0.05 mg/kg + GEN 10 mg/kg (400X; H&E). Hepatic parenchyma with neutrophilic inflammatory focus (arrow); **F**) Infected group/CAF 0.5 mg/kg + GEN 10 mg/kg (400X; H&E). Note hepatocytes with coagulative necrosis (arrow) and mild neutrophilic inflammatory focus (arrowhead); **G**) Infected group/CAF 5 mg/kg + GEN 10 mg/kg (400X; H&E). Note mild neutrophilic inflammatory focus (arrowhead) and Kupffer cell activation

Figure 7 shows the histological damage in animals subjected to treatments. Control uninfected plus untreated animals presented only discrete lymphocytic and neutrophilic infiltrates. Untreated *Salmonella*-infected mice exhibited moderate to intense, multifocal infiltrates composed of mononuclear lymphoplasmacytic cells and neutrophils. Sinusoidal capillaries were heavily infiltrated by neutrophils and activated, proliferating Kupffer cells. *Salmonella*-infected mice treated exclusively with gentamicin or caffeine exhibited moderated to intense, multifocal mononuclear lymphoplasmacytic and neutrophilic infiltrates, with sinusoidal capillaries infiltrated by activated, proliferating Kupffer cells. Mice co-administered 5 mg/kg caffeine and 10 mg/kg gentamicin displayed similar infiltrates, but only at minimal to mild intensity. By contrast, mice treated with 0.05 or 0.5 mg/kg caffeine exhibited liver lesions and infiltrates that were uniformly intense.

## Discussion

The U.S. Food and Drug Administration classify caffeine as Generally Recognized As Safe (GRAS) at concentrations up to 200 ppm (200 mg/L) [28]. Horringan et al. [29] showed that 100 µM caffeine (≈ 19.4 mg/L) in vitro approximates the plasma levels achieved through typical human consumption. In previous work, caffeine monotherapy reduced *S.* Typhimurium loads in peritoneal fluid, bloodstream, spleen, and liver of infected mice [24]. Previous checkerboard assays showed that 250 µg/ml caffeine halved gentamicin’s minimum inhibitory concentration against Gram-negative bacteria, indicating synergistic action [30]. Here, we investigated whether co-administration of caffeine and gentamicin enhances anti-*Salmonella* activity in a murine typhoid fever model. In vitro, caffeine had no direct antibacterial activity and did not potentiate gentamicin’s inhibitory effect at concentrations of 0.05–5 µg/ml. Although both caffeine and gentamicin are known to exert concentration- and time-dependent effects on cell viability [31, 32], neither agent alone nor their combination affected viability of uninfected primary murine peritoneal macrophages at any concentration tested.

Caffeine functions as a non-selective antagonist of adenosine receptors [33]. Adenosine is both an intra- and extracellular signaling molecule classified as a pre- and postsynaptic neuromodulator; its effects occur when it binds to one of four G protein–coupled receptors (A1, A2A, A2B, and A3) composed of α, β, and γ subunits [34, 35]. Binding to A1 and A3 receptors (generally inhibitory) decreases cyclic adenosine monophosphate (cAMP), whereas interaction with A2A and A2B receptors (generally excitatory) increases cAMP levels [33]. Caffeine can also modulate immune responses, since adenosine receptors are expressed on the surface of various immune cells [36]. By antagonizing A2A receptors, caffeine inhibits phosphodiesterase (PDE) activity and triggers protein kinase A (PKA) activation, ultimately suppressing pro-inflammatory cytokine production.

In *Salmonella*-infected macrophages, co-treatment with gentamicin (10 µg/ml) and low-dose caffeine (0.05–0.5 µg/ml) significantly reduced cell viability and led to increased intracellular CFU counts. This effect was not seen with 5 µg/ml caffeine plus 10 µg/ml gentamicin, indicating that only the lower caffeine concentrations antagonize gentamicin´s antibacterial activity. In vivo, combined treatment with caffeine (5 mg/kg) and gentamicin (10 mg/kg) lowered hepatic bacterial loads, attenuated inflammatory infiltrates and liver lesion severity. Extrapolating these animal regimens to human equivalent doses yielded 0.4 mg/kg and 0.81 mg/kg, respectively, corresponding to roughly 24.3 mg of caffeine and 48.6 mg of gentamicin for a 60-kg adult, which is reasonable. In mice receiving low-dose caffeine (0.05 and 0.5 mg/kg), gentamicin’s antibacterial efficacy was abolished, leading to higher CFU counts and more severe liver lesions in those groups. Taken together, the data obtained suggested a possible antagonistic interaction between caffeine and the antibiotic gentamicin in a dose-dependent manner. This hypothesis will be further investigated.

Caffeine’s anti-inflammatory effects are well documented and may explain the reduced neutrophil recruitment to the bloodstream and the decreased hepatic inflammatory infiltrates following *Salmonella* infection. In subpopulations of human monocyte-derived macrophages differentiated in the presence of macrophage colony-stimulating factor (M-CSF) and activated with LPS, caffeine suppressed TNF-α secretion and induced a significant increase in the expression of IL-6, IL-8, IL-1β, and NOD-like receptors [37]. In mice infected with *Listeria monocytogenes*, caffeine treatment reduced leukocyte infiltration and bacterial loads in the spleen and liver, and also decreased mRNA expression of IL-1β, IL-6, and inducible nitric oxide synthase (iNOS) [38]. We conclude that coadministration of gentamicin with caffeine reduces liver lesions through caffeine’s anti-inflammatory properties but impairs the antibiotic’s bacterial clearance – especially at lower caffeine doses – ultimately contributing to increased mortality in infected animals.
